# Electrical impedance tomography and its applicability in respiratory support in neonatal intensive care units: a protocol for a scoping review

**DOI:** 10.62675/2965-2774.20260412

**Published:** 2026-06-03

**Authors:** Marília Carvalho Borges, Ana Flávia Lozano Valadão Caserta, Suzana Cristina Almeida, Ingrid Guerra Azevedo, Vivian Mara Gonçalves de Oliveira Azevedo

**Affiliations:** 1 Universidade Federal de Uberlândia Faculdade de Educação Física e Fisioterapia Brazil Postgraduate Program in Physiotherapy, Faculdade de Educação Física e Fisioterapia, Universidade Federal de Uberlândia (UFU), Brazil.; 2 Universidade Federal de Uberlândia Faculdade de Educação Física e Fisioterapia Uberlândia MG Brazil Faculdade de Educação Física e Fisioterapia, Universidade Federal de Uberlândia - Uberlândia (MG), Brazil.; 3 Universidad Católica de Temuco Temuco Chile Universidad Católica de Temuco - Temuco, Araucania, Chile.

**Keywords:** Infant, Electrical impedance, Tomography, Pulmonary ventilation, Intensive care units

## Abstract

**Introduction:**

The neonatal intensive care unit employs advanced technologies to manage critical conditions. Electrical impedance tomography is a noninvasive, radiation-free imaging technique that provides real-time monitoring of pulmonary ventilation. The literature on electrical impedance tomography in the neonatal population is scattered and heterogeneous, justifying a scoping review.

**Objective:**

To map and synthesize the available evidence on the applicability of electrical impedance tomography in neonates receiving respiratory support in the neonatal intensive care unit.

**Methods:**

This protocol follows the steps described by Arksey and O’Malley as well as the recommendations of PRISMA-ScR and the Joanna Briggs Institute Manual for scoping reviews. The following databases will be used to guide searches: PubMed®, Embase, CINAHL, Web of Science, and Cochrane, with no date restrictions, in English, Spanish, and Portuguese. Two independent reviewers will screen, extract data, and assess the quality of eligible studies.

**Ethics and dissemination:**

Ethics approval is not required for this protocol and scoping review, as the study will rely exclusively on data from previously published research that has already obtained ethical approval. The results will be disseminated in a peer-reviewed scientific journal.

## INTRODUCTION

Life support in a neonatal intensive care unit (ICU) is essential to ensure the survival of newborns in critical condition.^(1)^ These units are equipped with advanced resources for continuous monitoring of premature infants or those with complications requiring intensive care.^(1)^ Adequate respiratory support is one of the main pillars of neonatal intensive care, being essential for monitoring and treating conditions such as apnea of prematurity, neonatal respiratory distress syndrome, and respiratory failure.^(2,3)^ A multidisciplinary approach, continuous monitoring of respiratory parameters, and the application of advanced technologies are essential for optimizing clinical outcomes, enabling timely and effective interventions.^(4,5)^

The introduction of new technologies in the neonatal ICU has transformed how healthcare professionals monitor and manage neonates receiving mechanical ventilation.^(6)^ Advanced tools, including electrical impedance tomography (EIT), ultrasound, and wireless monitors, have shown significant potential to complement the assessment of critically ill patients.^(7)^

Within this context of technological innovation, respiratory monitoring plays a key role in the care of newborns.^(8)^ This practice involves the continuous monitoring of parameters such as tidal volume, airway pressure, and ventilator-patient interaction, to optimize ventilation and ensure that the support provided is as adequate as possible.^(8)^ Devices that allow precise respiratory monitoring are especially important in neonates, whose immature and delicate lungs require careful observation to prevent complications.^(9)^

One recent technological innovation in respiratory monitoring is EIT. This noninvasive, radiation-free imaging technique employs low-frequency electrical currents to provide detailed images of lung ventilation distribution.^(10,11)^ In mechanically ventilated neonatal patients, EIT enables continuous monitoring of ventilation, facilitating precise adjustments of ventilatory parameters and early detection of complications such as atelectasis or pulmonary edema.^(12,13)^ Although EIT still has some limitations, such as limited spatial resolution and the need for precise calibration, it represents a promising advance in neonatal care, offering a safer and more effective method for assessing lung function and improving clinical outcomes.^(10,14)^

Despite this, the scientific literature on EIT is still scattered, with heterogeneous objectives and outcomes, particularly in neonatal populations. Existing reviews often combine multiple study designs, include animal research, or focus on pediatric patients or specific diagnoses, such as respiratory distress syndrome, making a robust quantitative synthesis unfeasible. Therefore, a scoping review is justified to map key concepts, identify gaps, and synthesize existing evidence on the use of EIT in neonates admitted to neonatal ICUs, particularly those under mechanical ventilation.

### Objective

To map and synthesize the available evidence on the applicability of EIT in neonates receiving respiratory support in the neonatal intensive care unit.

## METHODS

This protocol will serve as the basis for establishing the methodology for a scoping review to verify the applicability of EIT and its contribution to the management of mechanical ventilation in neonates admitted to the neonatal ICU. The review will be conducted in accordance with the recommendations of the international PRISMA-ScR10 guide and the Joanna Briggs Institute Reviewers Manual,^(15–18)^ and in accordance with the theoretical framework established by Arksey et al.^(19)^ Thus, the review will follow five steps: identification of the research question, identification of relevant studies, selection of studies, data analysis, synthesis, and presentation of data. In addition, the methodological quality of the selected studies will be assessed.

### Review registration

This review protocol has been registered with Open Science Framework (DOI 10.17605/OSF.IO/QFEGM).

### Review question

The main question that will guide this research is: "What are the applications of EIT in neonates receiving respiratory support in neonatal ICUs?"

1. The sub-questions of the research are:
2. What are the functional parameters for assessing lung ventilation distribution in neonates using EIT?
3. What are the possible adverse effects of using EIT?
4. What contributions can EIT offer in the assessment of ventilatory analysis in neonatology?
5. What are the benefits of EIT compared to other methods of ventilatory monitoring?

### Eligibility criteria

The eligibility criteria for article selection will be based on the Population-Concept-Context (PCC) framework, in which "P" refers to the population (newborns), "C" represents the concept of interest (EIT for respiratory monitoring), and "C" denotes the context (patients admitted to the neonatal ICU receiving respiratory support) (Table 1).

**Table 1:** Operational definitions

Element	Operational definition
P- Population	Newborns aged 0 to 28 days, including premature babies
C- Concept	Use of electrical impedance tomography for monitoring pulmonary ventilation
C- Context	Admission to the neonatal ICU with any type of respiratory support (invasive or noninvasive)

ICU - intensive care unit.

### Types of sources

We will include observational and experimental studies that used EIT to perform respiratory monitoring in neonates admitted to neonatal ICUs.

Studies conducted on animals, case studies, opinion articles, letters from specialists, and studies that include infants older than 28 days of age will not be included. Studies that do not provide clear data on the outcomes of interest will be excluded.

### Search strategy

The studies will be identified through searches of the following electronic databases: PubMed®, EMBASE, Cumulative Index to Nursing and Allied Health Literature (CINAHL), Web of Science, and the Cochrane Central Register of Controlled Trials based on the Population-Concept-Context (PCC). The detailed search strategies for each database are presented in table 1S (Supplementary Material).

For additional resources, we will search in the International Clinical Trials Registry Platform and the gray literature (ongoing studies not published in specialized conference proceedings, major clinical trial registries, and theses). In addition, the reference lists of the included articles will be screened to identify other eligible studies.

All searches will be limited to English, Spanish, and Portuguese, with no restrictions on publication date.

### Study selection

The studies identified through the search strategy will be imported into the Rayyan application^(20)^ for duplicate removal. Following this, two blinded investigators will independently screen the titles and abstracts to select studies for inclusion. Studies that meet the inclusion criteria will be selected for the review, while those that do not will be excluded. After initial screening based on the title and abstract, the full text will be read and re-applied to the inclusion and exclusion criteria by two blinded researchers. Only studies with consensus among the authors will be included. Disagreements will be resolved by a third investigator. The results of the database and other repository searches, as well as the study selection process, will be presented using a PRISMA-ScR flowchart.

### Data extraction and management

Two authors will independently extract data using a data extraction form that has been pre-tested and validated by the research team, and are presented in table 2S (Supplementary Material). When relevant data are unclear or not reported, we will contact the corresponding author of the publication for further details. The data will be organized into tables containing the key characteristics of the studies. The following will be presented: author and year of publication, study location, objective, study design, inclusion and exclusion criteria, interventions, and main results.

### Synthesis and presentation of data

The data from the reports will be organized into tables describing the important and relevant characteristics. An analysis of all content will be performed, with categories developed based on research questions. The results will then be compiled and presented, organized according to the elements relevant to addressing the research question.

### Quality assessment

Although scope reviews do not conventionally require a methodological assessment, we chose to include it in this protocol because EIT is increasingly used as a respiratory monitoring technology in neonates. Moreover, the available evidence on the topic is heterogeneous and scattered, encompassing various study designs and often small sample sizes.

A critical analysis of methodological quality will enable mapping the robustness of the evidence and identifying limitations that may affect the interpretation of results, thereby informing recommendations for future research and supporting transparent communication with professionals and decision-makers.

This assessment will not be used as a criterion for excluding studies, but rather as an additional resource for contextualizing the synthesis. To this end, a pilot test will be conducted with six studies to ensure consistency between the two reviewers responsible for the selection and data extraction process. They will then evaluate each eligible study for methodological quality before inclusion in the review.

Observational studies will be evaluated using the 2024 version of the Risk of Bias in Non-randomized Studies of Interventions (ROBINS-I) tool, as described in chapter 25 of the Cochrane Handbook for Systematic Reviews of Interventions.^(21)^ This tool assesses multiple domains, including risk of bias due to confounding, intervention classification, participant selection, deviations from intended interventions, missing data, outcome measurement, and selective reporting of results.^(21,22)^

Each domain has flag questions that are answered with "Yes," "Probably yes," "Probably no," "No," or "No information." The responses are classified by an algorithm into one of the following risk categories:

1. Low risk of bias (little or no concern about bias in the domain).
2. Moderate risk of bias (some concern about bias in the domain, but it is unclear whether there is an important risk of bias).
3. Serious risk of bias (the study has some important problems in the domain).
4. Critical risk of bias (the characteristics of the study give rise to a critical risk of bias, such that the result should generally be excluded from evidence syntheses).

For non-randomized observational studies without a control group, methodological quality will be assessed using the Methodological Index for Non-Randomized Studies (MINORS).^(23)^ This validated instrument is specifically designed to evaluate the methodological quality of non-comparative studies and includes criteria such as a clearly stated aim, inclusion of consecutive patients, prospective data collection, appropriate endpoints, unbiased assessment of outcomes, and adequate follow-up. For randomized studies, the Cochrane Risk of Bias Tool for Randomized Trials (RoB 2) version 2019, described in chapter 8 of the Cochrane Handbook for Systematic Reviews of Interventions,^(21)^ will be used. RoB 2 covers five domains of bias: bias arising from the randomization process, bias due to deviations from the intended interventions, bias due to lack of outcome data, bias in outcome measurement, and bias in the selection of reported outcomes.^(21,22)^

The tool includes algorithms that map to screening questions that are answered with "Yes," "Probably yes," "Probably no," "No," or "No information." Thus, risk assessment is performed at the domain level and globally. The possible assessments of risk of bias are:

1. Low risk of bias (low risk of bias for all domains).
2. Some concerns ("some concerns" in at least one domain, but no high risk of bias for any domain).
3. High risk of bias (high risk of bias in at least one domain, or the study is judged to have some concerns for multiple domains in a way that substantially reduces confidence in the result).

We will report these assessments for each study in the "Risk of bias" tables in the review and discuss the impact of methodological quality on the results. Any disagreement will be resolved by consultation with a fourth author.

## ETHICS AND DISSEMINATION

Ethical approval is not required from the institution's research ethics committee, as this exploratory review does not involve the collection of primary data. The results of this exploratory review will be presented at scientific conferences and published in a peer-reviewed scientific journal, in accordance with PRISMA-ScR guidelines.

## Data Availability

No datasets were generated or analyzed during the current study. This protocol has been registered in the Open Science Framework (OSF), and all relevant data will be obtained from publicly available sources and reported in the final review.
