# Supplementary material for: Electrical impedance tomography and its applicability in respiratory support in neonatal intensive care units: a protocol for a scoping review
Source: Crit Care Sci. 2026 Jun 3;38:e20260412. doi: 10.62675/2965-2774.20260412 (PMC13399250; doi:10.62675/2965-2774.20260412)
Supplement: Supplementary Material [file 2965-2774-ccsci-38-e20260412-suppl01.pdf]

Critical Care Science
https://doi.org/10.62675/2965-2774.20260412

SUPPLEMENTARY MATERIAL

Electrical impedance tomography and its applicability in respiratory support in neonatal intensive care units: a protocol for a scoping review

Marília Carvalho Borges[1], Ana Flávia Lozano Valadão Caserta[2], Suzana Cristina Almeida[2], Ingrid Guerra Azevedo[3], Vivian Mara Gonçalves de Oliveira Azevedo[1,2]

Table 1S - Search for each individual database

Database		Search strategy
PubMed®		"Infant, Newborn"[Mesh] OR (Infant, Newborn) OR (Infants, Newborn) OR (Newborn Infant) OR (Newborn Infants) OR Neonate OR Neonates OR Newborns OR Newborn
	AND	"Tomography"[Mesh] OR Tomography OR Tomographies
	AND	"Electric Impedance"[Mesh] OR (Electric Impedance) OR (Impedance, Electric) OR Impedance OR (Electrical Impedance) OR (Impedance, Electrical) OR (Electric Resistance) OR (Resistance, Electric) OR (Electrical Resistance) OR (Resistance, Electrical) OR (Bioelectrical Impedance) OR (Impedance, Bioelectrical) OR (Biolectric Impedance) OR (Impedance, Biolectric) OR (Ohmic Resistance) OR (Ohmic Resistances) OR (Resistance, Ohmic) OR (Resistances, Ohmic)
	AND	"Intensive Care Units, Neonatal"[Mesh] OR (Intensive Care Units, Neonatal) OR (Newborn Intensive Care Units) OR (Newborn Intensive Care Units (NICU)) OR (Neonatal Intensive Care Units) OR (Newborn Intensive Care Unit) OR (Neonatal Intensive Care Unit) OR (ICU, Neonatal) OR (ICUs, Neonatal) OR (Neonatal ICUs) OR (Newborn ICU) OR (ICU, Newborn) OR (ICUs, Newborn) OR (Newborn ICUs) OR (Neonatal ICU)
Web of Science		"Infant, Newborn" OR "Infant, Newborn" OR "Infants, Newborn" OR "Newborn Infant" OR "Newborn Infants" OR Neonate OR Neonates OR Newborns OR Newborn
	AND	"Tomography" OR Tomography OR Tomographies
	AND	"Electric Impedance" OR "Electric Impedance" OR "Impedance, Electric" OR Impedance OR "Electrical Impedance" OR "Impedance, Electrical" OR "Electric Resistance" OR "Resistance, Electric" OR "Electrical Resistance" OR "Resistance, Electrical" OR "Bioelectrical Impedance" OR "Impedance, Bioelectrical" OR "Biolectric Impedance" OR "Impedance, Biolectric" OR "Ohmic Resistance" OR "Ohmic Resistances" OR "Resistance, Ohmic" OR "Resistances, Ohmic"
	AND	"Intensive Care Units, Neonatal" OR "Intensive Care Units, Neonatal" OR "Newborn Intensive Care Units" OR "Newborn Intensive Care Units (NICU)" OR "Neonatal Intensive Care Units" OR "Newborn Intensive Care Unit" OR "Neonatal Intensive Care Unit" OR "ICU, Neonatal" OR "ICUs, Neonatal" OR "Neonatal ICUs" OR "Newborn ICU" OR "ICU, Newborn" OR "ICUs, Newborn" OR "Newborn ICUs" OR "Neonatal ICU"

Continue...

This is an open access article under the CC BY license (https://creativecommons.org/licenses/by/4.0/).

...continuation

Scopus		"Infant, Newborn" OR "Infant, Newborn" OR "Infants, Newborn" OR "Newborn Infant" OR "Newborn Infants" OR Neonate OR Neonates OR Newborns OR Newborn
	AND	"Tomography" OR Tomography OR Tomographies
	AND	"Electric Impedance" OR "Electric Impedance" OR "Impedance, Electric" OR Impedance OR "Electrical Impedance" OR "Impedance, Electrical" OR "Electric Resistance" OR "Resistance, Electric" OR "Electrical Resistance" OR "Resistance, Electrical" OR "Bioelectrical Impedance" OR "Impedance, Bioelectrical" OR "Biolectric Impedance" OR "Impedance, Biolectric" OR "Ohmic Resistance" OR "Ohmic Resistances" OR "Resistance, Ohmic" OR "Resistances, Ohmic"
	AND	"Intensive Care Units, Neonatal" OR "Intensive Care Units, Neonatal" OR "Newborn Intensive Care Units" OR "Newborn Intensive Care Units (NICU)" OR "Neonatal Intensive Care Units" OR "Newborn Intensive Care Unit" OR "Neonatal Intensive Care Unit" OR "ICU, Neonatal" OR "ICUs, Neonatal" OR "Neonatal ICUs" OR "Newborn ICU" OR "ICU, Newborn" OR "ICUs, Newborn" OR "Newborn ICUs" OR "Neonatal ICU"
Embase		'newborn'/exp OR newborn OR 'animals, newborn' OR 'child, newborn' OR 'full term infant' OR 'human neonate' OR 'human newborn' OR 'infant, newborn' OR 'neonatal animal' OR 'neonate' OR 'neonate animal' OR 'neonatus' OR 'newborn animal' OR 'newborn animals' OR 'newborn baby' OR 'newborn child' OR 'newborn infant' OR 'newly born animal' OR 'newly born baby' OR 'newly born child' OR 'newly born infant' OR 'newborn'
	AND	'electrical impedance tomograph'/exp OR 'electrical impedance tomography device' OR 'Enlight 1810' OR 'Enlight 2100' OR 'PhysioFlow' OR 'PulmoVista 500' OR 'thoracic electric impedance tomograph' OR 'thoracic electrical bioimpedance device' OR 'thoracic electrical bioimpedance system' OR 'thoracic electrical bioimpedance system, segmentographic' OR 'thoracic electrical bioimpedance system, tomographic' OR 'electrical impedance tomograph'
	AND	'computer-assisted impedance tomography'/exp OR 'electrical impedance tomography' OR 'impedance computer tomography' OR 'impedance tomography' OR 'tomography, computer-assisted impedance' OR 'computer-assisted impedance tomography'
	AND	'neonatal intensive care unit'/exp OR 'intensive care unit, newborn' OR 'intensive care units, neonatal' OR 'neo-natal intensive care unit' OR 'neonatal ICU' OR 'neonatal ICUs' OR 'neonatal intensive care department' OR 'newborn ICU' OR 'newborn ICUs' OR 'newborn intensive care department' OR 'newborn intensive care unit' OR 'NICU (neonatal)' OR 'NICUs (neonatal)' OR 'neonatal intensive care unit'
Cochrane		Infant, Newborn
	AND	Tomography
	AND	Electric Impedance
	AND	Intensive Care Units, Neonatal
CINAHL		Infant, Newborn
	AND	Tomography
	AND	Electric Impedance
	AND	Intensive Care Units, Neonatal

CINAHL: Cumulative Index to Nursing and Allied Health Literature.

Table 2S - Data extraction form

**Study details**	
Author(s)	
Publication year	
Objectives	
Design	
Key findings	
**Population**	
Population characteristics (age)	
Sample size	
**Concept:**	
Measures of electrical impedance tomography	
Purpose of use	
Comparator (if applicable)	
Intervention (if applicable)	
**Context**	
Research setting	
Type of respiratory support	
